# A serial mediation model of patient safety climate on nurses' compliance with standard precautions: the roles of infection prevention climate and attitude

**DOI:** 10.3389/fpubh.2025.1673026

**Published:** 2025-09-18

**Authors:** Wenjing Jiang, Li Cao, Qiurun Liu, Ping Zhou, Anna Dai, Xiaoyu Liao, Juan Tang

**Affiliations:** ^1^Department of Hospital Infection Management, Zigong First People's Hospital, Zigong, China; ^2^Department of Nursing, Sichuan Vocational College of Health and Rehabilitation, Zigong, China; ^3^Department of Gastrointestinal Surgery, West China Tianfu Hospital, Sichuan University, Chengdu, China; ^4^Department of Infectious Diseases, Zigong First People's Hospital, Zigong, China

**Keywords:** patient safety climate, infection prevention, standard precautions, compliance, social cognitive theory, serial mediation

## Abstract

**Objectives:**

This study tested a serial mediation model, based on Social Cognitive Theory (SCT), to explore how patient safety climate is associated with nurses' compliance via infection prevention climate (IPC) and standard precautions attitude.

**Methods:**

A cross-sectional survey was conducted among 913 registered nurses from 34 hospitals in China using validated instruments. Data were analyzed using PROCESS macro (Model 6) with 5,000 bootstrap samples, adjusting for age, gender, education, professional title, hospital level, and infection control training frequency.

**Results:**

Patient safety climate had a significant total effect on compliance (βPSC → COMP(total) = 0.551, *p* < 0.001), but the direct effect became non-significant after including mediators (βPSC → COMP = −0.034, *p* = 0.378), indicating full mediation. This suggests that patient safety climate was not directly associated with compliance but exerted its influence entirely through infection prevention climate and attitudes, highlighting an indirect yet substantial pathway. All three indirect paths were significant: via infection prevention climate alone (βPSC → IPC → COMP = 0.138), via attitude alone (βPSC → ATT → COMP = 0.102), and via both in sequence [βPSC → IPC → ATT → COMP = 0.346, 95% CI (0.272, 0.426)], with the sequential pathway explaining 59.0% of the total indirect effect. In practical terms, attitude showed the strongest predictive power, with a standardized coefficient of βATT → COMP = 0.666, indicating a clinically meaningful impact on compliance behavior.

**Conclusion:**

Patient safety climate indirectly enhances compliance through departmental climate and individual attitudes. Interventions targeting both organizational climate and individual beliefs may strengthen compliance with standard precautions in clinical practice.

## 1 Introduction

Patient safety is widely recognized as a cornerstone of healthcare quality—a principle that gained renewed prominence during the COVID-19 pandemic, when infection control became critically important (1–3). Patient safety climate—commonly defined as collective perceptions regarding leadership commitment, open communication, and organizational learning related to safety—has been consistently associated with improved adherence to standard precautions and reductions in healthcare-associated infections (HAIs) (4, 5). For instance, a multicenter observational study found that a positive patient safety climate was significantly associated with higher compliance with standard precautions and improved HAI outcomes (6).

Despite substantial evidence linking patient safety climate to behavior, the underlying mechanisms remain insufficiently explored—especially those that integrate organizational, departmental, and individual determinants (7–14). Guided by Bandura's Social Cognitive Theory (SCT), which emphasizes the interaction between environment, cognition, and behavior in shaping human actions (13), this study explores how patient safety climate as an organizational-level climate factor may influence individual compliance behaviors through intermediary cognitive and contextual pathways (7, 14).

To operationalize these theoretical foundations, this study proposes a multilevel serial mediation model illustrating how patient safety climate—an organizational-level climate factor—influences nurses' compliance with standard precautions through the sequential mediators of infection prevention climate (IPC) and individual attitudes. Prior research suggests that organizational safety climate can cascade downward, shaping departmental climates through leadership emphasis, policy enforcement, and allocation of infection control resources (8, 10, 12). This theoretical framework supports our hypothesis that patient safety climate associates with infection prevention climate. Patient safety climate reflects the macro-level organizational context, which is theoretically associated with the meso-level departmental environment—operationalized as infection prevention climate (15, 16)—that in turn correlates with nurses' personal attitudes toward standard precautions, with these factors collectively associated with compliance behavior (17).

This conceptualization is supported by multilevel organizational theories, particularly the work of Kozlowski and Klein, who emphasized that organizational behaviors emerge from nested contextual layers and operate through cross-level influences (18). Their model highlights that macro-level climates influence micro-level behaviors via intermediate levels, necessitating integrated analyses that capture such cross-level transmission (18). Applying this framework to infection control is innovative and addresses a persistent gap in the literature, which tends to examine organizational climate and individual attitudes separately, often neglecting the influence of intermediate departmental climates.

Among departmental-level factors, infection prevention climate was identified as a key meso-level mediator due to its direct relevance to infection control practices (19). Unlike general department characteristics such as workload or team cohesion, infection prevention climate specifically captures healthcare workers' shared perceptions of norms, available resources, and leadership commitment to infection control (19, 20). It thus, functions as a crucial bridge connecting the broader organizational safety climate to individual cognitive and behavioral outcomes (7, 19, 21).

Recent empirical studies within infection prevention support this selection (19, 22). For example, Rumintang Marito and Widianto demonstrated that infection prevention climate significantly mediates the relationship between workplace safety and patient-centered care, and also between risk mitigation and patient-centered care, underscoring its central role in linking organizational climate to clinical behaviors and outcomes (19). Similarly, Kim and Lee (22) found that organizational climate for infection control was closely linked with emergency department nurses' compliance with standard precautions, reinforcing its role as a critical organizational-level predictor in the pathway from individual cognitive factors to behavior.

When individual attitudes are considered, compliance behavior is more strongly predicted, consistent with the Theory of Planned Behavior (TPB) and SCT frameworks (21, 23, 24). Attitudes influence intentions, which ultimately guide behavior. Specifically in infection control contexts, positive attitudes toward standard precautions have demonstrated strong predictive power for compliance behaviors (21, 22).

These constructs align with the principles of SCT, in which environmental inputs shape cognition and subsequently influence behavior (13, 24, 25). Although such serial mediation frameworks are well-established in industrial safety literature, their application in healthcare—particularly infection control—remains limited (26, 27). For instance, a recent study found that safety-specific leadership influenced employee safety behavior via team climate and motivation (26). Building on this rationale, the present study investigates how organizational safety climate is transmitted into individual compliance through departmental and cognitive mechanisms.

**Figure 1:**
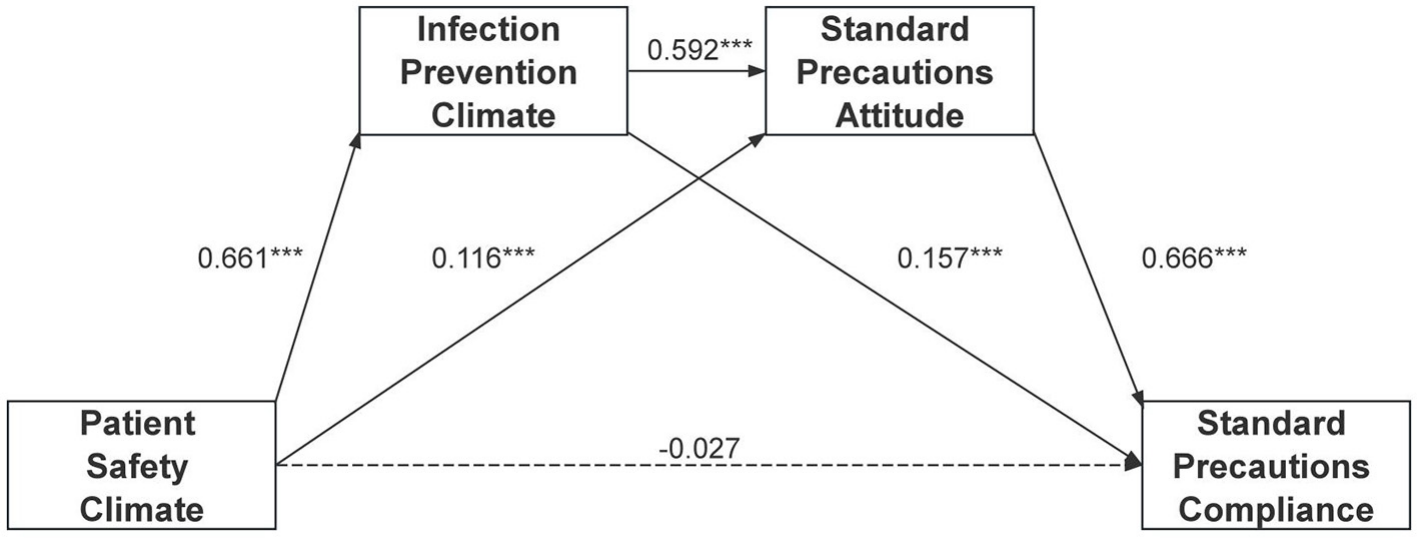
Results of multiple mediation analysis. Standardized coefficients are presented. Standardized association pathways: PSC → IPC: β = 0.661, *p* < 0.001; IPC → ATT: β = 0.592, *p* < 0.001; ATT → COMP: β = 0.666, *p* < 0.001. ***p < 0.001. Full model-comparison results in Supplementary Table S1.

Accordingly, we tested a serial mediation model in which patient safety climate influences nurses' compliance with standard precautions through infection prevention climate and standard precautions attitude, both as parallel and as sequential mediators. The proposed model structure is shown in Figure 1, and mediation effects were examined using bootstrap resampling.

### 1.1 Research hypotheses

Building upon these theoretical and empirical premises, we propose the following hypotheses:

- H1: Patient safety climate positively predicts infection prevention climate.
- H2: Both patient safety climate and infection prevention climate positively influence standard precautions attitude.
- H3: Standard precautions attitude positively predicts compliance behavior.
- H4: Infection prevention climate mediates the relationship between patient safety climate and compliance.
- H5: Standard precautions attitude mediates the relationship between patient safety climate and compliance.
- H6: A sequential serial mediation exists whereby patient safety climate influences compliance through infection prevention climate and subsequently through attitude.

By empirically testing these hypotheses, this study aims to advance the theoretical application of SCT in infection control by integrating organizational, departmental, and individual determinants into a unified serial mediation model. The findings are expected not only to clarify the mechanisms linking institutional climate to frontline behaviors, but also to provide actionable insights for designing multilevel interventions to improve standard precautions compliance.

## 2 Methods

### 2.1 Study design

A cross-sectional, descriptive correlational study was conducted to examine the hypothesized serial mediation model involving patient safety climate, infection prevention climate, standard precautions attitude, and standard precautions compliance. The study was reported in accordance with the Strengthening the Reporting of Observational Studies in Epidemiology (STROBE) guidelines.

### 2.2 Participants

The study targeted clinical nurses and nurse managers working in Chinese hospitals. Inclusion criteria were: (1) registered nurses currently working in a clinical department (including both frontline clinical nurses and nurse managers); (2) at least 6 months of continuous employment at their current hospital; and (3) voluntary agreement to participate. Nurses who were on leave or working exclusively in non-clinical positions (e.g., research or education departments) were excluded.

A total of 947 responses were collected using online questionnaires. After removing incomplete or invalid submissions, 913 valid responses were retained, resulting in a valid response rate of 96.4%. This sample size exceeded the recommended minimum for mediation analysis, supporting the statistical power and stability of the model estimates.

### 2.3 Data collection

A convenience sampling strategy was employed. Data were collected using an online questionnaire administered via Wenjuanxing (www.wjx.cn), a widely used survey platform in China. Recruitment was facilitated through the National Nursing Quality Control Center: electronic invitations were first distributed to provincial directors of nursing quality control via WeChat, who then disseminated the survey link to hospital-level nurse managers and their internal nurse WeChat groups. The first page of the online survey described the purpose, voluntary nature, and anonymity of the study. Participants indicated informed consent by clicking “Agree” to proceed. No personally identifiable information was collected. Participants were allowed to exit the survey at any time. The data collection period lasted from April 15 to May 30, 2025. All data were stored on encrypted institutional servers and were used solely for academic research.

### 2.4 Ethical considerations

The study protocol was reviewed and approved by the Ethics Committee of Zigong First People's Hospital (Approval No. 2025-045). Participation was entirely voluntary, and informed consent was obtained electronically. Data collection adhered to the ethical principles of the Declaration of Helsinki and ensured confidentiality and anonymity throughout.

### 2.5 Measures

The survey included scales or subscales to measure the key study variables. The total mean score for each scale was computed, with a higher score indicating a higher level of the construct. Also, the survey collected respondents' general information.

#### 2.5.1 Outcome variables

Standard precautions compliance was measured using four items of standard precautions questionnaire, which was developed by Michinov et al. (17) and translated by Huang et al. (28). An example item for compliance is “I comply with the hospital's standard precautions protocols regardless of time constraints.” Responses were measured on a 5-point scale ranging from 1 (strongly disagree) to 5 (strongly agree) depending on the item, and the total mean score was calculated. Cronbach's alpha for the scale was 0.899 for the study sample.

#### 2.5.2 Predictor variables

Patient safety climate was measured using the Hospital Survey on Patient Safety Culture 2.0 (HSOPSC 2.0) originally developed by the Agency for Healthcare Research and Quality (15) and translated into a 32-item Chinese version and then validated by Yin et al. (29). An example item is “This unit regularly reviews work processes to determine if changes are needed to improve patient safety.” Responses were measured on a 5-point scale ranging from 1 (strongly disagree or never) to 5 (strongly agree or always) depending on the item, and the total mean score was calculated. Cronbach's alpha for the scale was 0.912 for the study sample.

#### 2.5.3 Mediating variables

Infection prevention climate was measured using the Leading a Culture of Quality for Infection Prevention originally developed by Pogorzelska-Maziarz et al. (16) and translated into a 15-item Chinese version and then validated by Li et al. (30). An example item is “The HAI prevention goals and strategic plan of our organization are clear and well-communicated.” Responses were measured on a 5-point scale ranging from 1 (strongly disagree) to 5 (strongly agree) depending on the item, and the total mean score was calculated. Cronbach's alpha for the scale was 0.965 for the study sample.

Standard precautions attitude was also measured using three items of standard precautions questionnaire (28). An example item for attitude is “If I follow the standard precautions protocol, I will protect my patients from infection.” Responses were measured on a 5-point scale ranging from 1 (strongly disagree) to 5 (strongly agree) depending on the item, and the total mean score was calculated. Cronbach's alpha for the scale was 0.986 for the study sample.

#### 2.5.4 Covariates

Age, gender, educational background, professional title, hospital level, and frequency of infection control training within the past 6 months were included as covariates. These variables were selected based on prior literature linking them to compliance behaviors and organizational perceptions (5, 11, 21).

### 2.6 Data analysis

All data were analyzed using IBM SPSS Statistics version 27.0 and the PROCESS macro version 4.1 developed by Hayes (31). Descriptive statistics were used to summarize demographic and professional characteristics. Pearson correlation coefficients were computed to examine the associations between the key study variables.

To test the hypothesized serial mediation model, PROCESS Model 6 was employed with 5,000 bootstrap resamples and a 95% confidence interval. In this model, patient safety climate served as the independent variable, standard precautions compliance as the dependent variable, and infection prevention climate and standard precautions attitude as sequential mediators. Both direct and indirect effects were estimated, and indirect effects were considered significant when the 95% bootstrap confidence intervals did not include zero. Standardized coefficients were reported for all paths to facilitate interpretation. This approach allowed for a clear assessment of how patient safety climate influences compliance both directly and indirectly through the proposed sequential mediators.

## 3 Results

### 3.1 Participant characteristics

A total of 913 valid responses were included in the analysis. The mean age of participants was 33.0 years (SD = 7.6). Most were female (91.2%) and held a bachelor's degree (72.2%). More than half of participants (50.2%) had 5–14 years of working experience, and 28.5% had prior infection control experience. Only 17.0% were currently working in an intensive care unit (ICU). Detailed demographic and professional characteristics are shown in Table 1.

**Table 1:** Demographic and professional characteristics of participants (*N* = 913).

**Variable**	***n* (%)**
Age (years), M ± SD	33.0 ± 7.6
**Gender**	
Male	80 (8.8%)
Female	833 (91.2%)
**Working years**	
≤ 5 years	213 (23.3%)
5–14 years	458 (50.2%)
15–24 years	157 (17.2%)
≥25 years	85 (9.3%)
**Educational background**	
Associate degree or below	237 (26.0%)
Bachelor's degree	659 (72.2%)
Master's degree or above	17 (1.9%)
**Managerial position**	
No	815 (89.3%)
Yes	98 (10.7%)
**Professional title**	
None	73 (8.0%)
Junior	407 (44.6%)
Intermediate	349 (38.2%)
Senior	84 (9.2%)
**Currently working in ICU**	
No	758 (83.0%)
Yes	155 (17.0%)
**Hospital level**	
Primary or undefined	55 (6.0%)
Secondary-level hospital	279 (30.6%)
Tertiary-level hospital	579 (63.4%)
**Infection control training (past 6 months)**	
0	51 (5.6%)
1–2 times	295 (32.3%)
3–4 times	223 (24.4%)
≥5 times	344 (37.7%)

M, mean; SD, standard deviation; ICU, intensive care unit.

### 3.2 Descriptive statistics and correlations

Descriptive statistics and Pearson correlations for the study variables are presented in Table 2. Patient safety climate was significantly and positively correlated with infection prevention climate (*r* = 0.670, *p* < 0.01), standard precautions attitude (*r* = 0.511, *p* < 0.01), and standard precautions compliance (*r* = 0.417, *p* < 0.01). Standard precautions attitude demonstrated the strongest correlation with compliance (*r* = 0.757, *p* < 0.01).

**Table 2:** Descriptive statistics and Pearson correlations (*r*) for study variables.

**Variable**	**M (SD)**	**1**	**2**	**3**	**4**
1. Patient safety climate	3.82 (0.49)	1			
2. Infection prevention climate	3.97 (0.60)	0.670^**^	1		
3. Standard precautions attitude	4.39 (0.63)	0.511^**^	0.667^**^	1	
4. Standard precautions compliance	4.22 (0.65)	0.417^**^	0.583^**^	0.757^**^	1

r, Pearson correlation coefficient; M, mean; SD, standard deviation.

^**^p <0.01.

### 3.3 Mediation model

PROCESS macro (Model 6) was used to examine the serial mediation effect of infection prevention climate and standard precautions attitude. The total association between patient safety climate and standard precautions compliance was significant [βPSC → COMP(total) = 0.551, SE = 0.041, 95% CI (0.471, 0.632), *p* < 0.001]. However, the direct relationship became non-significant after including the mediators (βPSC → COMP|IPC,ATT = −0.034, SE = 0.039, *p* = 0.378), indicating full mediation.

Patient safety climate demonstrated a robust association with infection prevention climate (βPSC → IPC = 0.661, *p* < 0.001), accounting for 43.7% of variance in IPC scores—the strongest path in our model. Model comparisons confirmed the theoretical necessity of including PSC, as the constrained model showed markedly poorer fit, with substantial AIC deterioration and a 37.2% reduction in IPC variance explained (Supplementary Table S1).

The total indirect association was significant [βIndirect(total) = 0.586, SE = 0.049, 95% CI (0.495, 0.687), *p* < 0.001]. Among the three specific indirect paths: the path through infection prevention climate alone (Ind1: X → M1 → Y) was significant [βPSC → IPC → COMP = 0.138, SE = 0.030, 95% CI (0.078, 0.199), *p* < 0.001]; The path through standard precautions attitude alone (Ind2: X → M2 → Y) was also significant [βPSC → ATT → COMP = 0.102, SE = 0.029, 95% CI (0.047, 0.161), *p* < 0.001]; The sequential path through both mediators (Ind3: X → M1 → M2 → Y) showed the strongest effect [βPSC → IPC → ATT → COMP = 0.346, SE = 0.039, 95% CI (0.272, 0.426), *p* < 0.001).

Standardized coefficients of the final model showed that infection prevention climate (βIPC → COMP = 0.157) and standard precautions attitude (βATT → COMP = 0.666) were strong predictors of compliance, while the direct effect of patient safety climate was not significant after controlling for mediators (βPSC → COMP|IPC,ATT = −0.026).

Contrast analyses between indirect paths revealed that the sequential path (Ind3) was significantly stronger than both the single-mediator paths (Contrast 2 and 3, *p* < 0.001). No significant difference was observed between the two single-mediator paths (Contrast 1: βInd1 vs. Ind2 = 0.035, *p* = 0.402).

A visual representation of the final model is provided in Figure 1, and detailed coefficients are summarized in Table 3.

**Table 3:** Multilevel mediation analysis of patient safety climate effects on standard precaution compliance.

**Effect type**	**Path**	**β (SE)**	**95% CI**	**Std. β**	***p-*value**
Total effect	X → Y	0.551 (0.041)	[0.471, 0.632]	0.415	<0.001
Direct effect	X → Y (controlling M1, M2)	−0.034 (0.039)	[−0.111, 0.042]	−0.026	0.378
Total indirect effect	All paths	0.585 (0.049)	[0.495, 0.687]	0.441	<0.001
**Specific indirect effects**					
Path 1	X → M1 → Y	0.138 (0.030)	[0.078, 0.199]	0.104	<0.001
Path 2	X → M2 → Y	0.102 (0.029)	[0.047, 0.161]	0.077	<0.001
Path 3	X → M1 → M2 → Y	0.346 (0.039)	[0.272, 0.426]	0.260	<0.001
**Contrast effects**					
Path 1 vs. Path 2	M1 → Y vs. M2 → Y	0.035 (0.046)	[−0.056, 0.124]	0.027	0.402
Path 1 vs. Path 3	M1 → Y vs. M1 → M2 → Y	−0.208 (0.049)	[−0.307, −0.115]	−0.157	<0.001
Path 2 vs. Path 3	M2 → Y vs. M1 → M2 → Y	−0.243 (0.055)	[−0.353, −0.140]	−0.183	<0.001

X = Patient Safety Climate, M1 = Infection Prevention Climate, M2 = Standard Precautions Attitude, Y = Standard Precautions Compliance. Bootstrapping samples = 5,000. Structural path coefficients are summarized in Figure 1; full parameter estimates and model comparison (excluding PSC vs. final) in Supplementary Table S1.

## 4 Discussion

This study provides empirical validation of a multilevel serial mediation model grounded in Bandura's SCT. The model shows that the relationship between patient safety climate and nurses' compliance is fully mediated by infection prevention climate and individual attitudes, aligning with our hypotheses. This provides robust support for a multi-level cognitive-environmental transmission mechanism, emphasizing the importance of addressing both departmental climate and individual attitudes in infection control interventions.

The absence of a direct effect of patient safety climate on compliance after including mediators (βPSC → COMP|IPC,ATT = −0.034, *p* = 0.378) confirms a complete mediation model. This is consistent with Griffin and Neal's theoretical model, which posits that distal organizational factors correlate with safety behavior indirectly through proximal motivational or cognitive mechanisms (32). Recent empirical work by Hessels et al. (6) corroborates this indirect pathway, showing that patient safety climate predicts compliance-related outcomes through its influence on intermediate behavioral drivers. Our model explicitly demonstrates that patient safety climate—a macro-level construct—affects behavior only via meso-level (infection prevention climate) and micro-level (attitude) pathways, reinforcing the layered ecological structure of behavioral development in healthcare.

The sequential mediation pathway [βPSC → IPC → ATT → COMP = 0.346, 95% CI (0.272, 0.426)] emerged as the most influential mechanism, which was significantly stronger than either of the single-mediator paths (Contrast 2 and 3, *p* < 0.001). This finding underscored that organizational influence on frontline behavior was transmitted through layered, cascading processes involving social norms and cognitive interpretations, rather than being direct or linear (13, 24).

The indirect relationship through infection prevention climate alone was substantial (βPSC → IPC → COMP = 0.138). This validates its role as a cross-level transmission mechanism. Infection prevention climate, as a meso-level construct, captures shared unit-level perceptions of infection control practices, leadership emphasis, and resource allocation (6). The strength of the PSC → IPC pathway (β=0.661) exceeds typical leadership effects in healthcare settings (β ≈ 0.35–0.45), highlighting its critical role in translating organizational safety priorities into unit-level norms (6). Practically, healthcare institutions can implement leadership-led safety rounds to reinforce visible commitment to infection prevention; foster departmental peer-feedback sessions to shape shared norms; and embed infection control principles into routine nursing briefings to sustain positive attitudes. These interventions exemplify the specific, actionable strategies implied by our model (33, 34).

From a practical standpoint, the magnitude of the effect sizes demonstrates their clinical relevance. Specifically, the standardized coefficient for standard precautions attitude (βATT → COMP = 0.666) indicates that a one–standard deviation increase in attitude is associated with a 0.666 SD increase in compliance—a large and meaningful effect. Similarly, a one–standard deviation improvement in infection prevention climate (βIPC → COMP = 0.157) corresponds to a 0.157 SD increase in compliance behavior. Together, these findings show that both individual cognitive factors and departmental environmental conditions have quantifiable impacts on frontline adherence.

Importantly, our findings clarify the distinction between infection prevention climate (IPC) and the broader organizational climate. While both are safety-related, IPC exerts a more proximal and behaviorally specific influence. For instance, Rozenbojm et al. demonstrated that was a significant predictor of nurses' compliance with facial protective equipment, emphasizing the behavior-specific nature of localized safety climates (35). Similarly, a pivotal intervention study by Larson et al. showed that targeted improvements in infection-related climate were associated with increased hand hygiene and reduced nosocomial infections, highlighting the practical impact of unit-level climate adjustments on measurable behaviors (36).

Beyond numerical effect sizes, IPC represents a qualitatively distinct intervention target compared with individual attitudes. While attitude reflects personal conviction, IPC captures shared social norms, leadership emphasis, and collective practices, making it a lever for systemic change. Thus, even when its standardized coefficient is smaller, the strategic importance of IPC for sustainable infection control should not be underestimated.

Standard precautions attitude also served as a significant mediator (βPSC → ATT → COMP = 0.102), consistent with the Theory of Planned Behavior (TPB) and the Social Cognitive Theory (SCT) proposition that cognition mediates environmental influence on behavior (37, 38). Nurses who believe in the efficacy and necessity of standard precautions are more likely to adhere to them, regardless of organizational context (37, 39). This emphasizes that favorable climate alone is insufficient; internalization of safety values and personal conviction remain essential (37, 38).

Notably, standard precautions attitude also acted as the final pathway in the sequential mediation chain, indicating that any upstream improvements in IPC must be translated into cognitive commitment to achieve behavioral effects (40, 41). This finding supports integrated interventions that not only change systems but also shape individual-level psychological readiness (41, 42).

Our findings carry several practical implications for healthcare administrators and infection control leaders. First, organizational investment in unit-level IPC—through leadership engagement, resource visibility, and clear communication—may yield disproportionate returns in behavioral compliance. For example, real-time dashboards monitoring personal protective equipment usage and daily safety briefings can strengthen perceptions of the local safety climate. Second, attitude-based interventions, such as immersive VR simulations and peer modeling, can consolidate IPC effects by reinforcing personal relevance and emotional salience of standard precautions (43). This dual-targeted approach reflects evidence that multi-component interventions are more effective than isolated strategies, especially in complex hospital systems.

Moreover, from a health system perspective, our finding showed that compliance behavior arose from a serial transmission of organizational signals through departmental and cognitive filters. This underscored the need for cross-level alignment in safety strategies. Accreditation standards and infection control evaluations should consider including unit-level climate metrics as quality indicators. Allocating at least 15% of infection control training budgets to team-based climate-building initiatives may enhance the sustainability of compliance gains.

Our study was conducted in Chinese hospitals, where healthcare hierarchies tend to be more pronounced and collectivist values are culturally reinforced. Compared to Western findings where individual-level attitudes may dominate behavioral variance, our results show a stronger meso-level influence from IPC (βIPC → COMP = 0.157 vs. βATT → COMP = 0.666), indicating that in high power distance contexts and tightly coupled work teams, social norms and departmental leadership may play a larger role in behavior regulation than individual cognition alone (44). Such cultural nuances should be considered in intervention design (45). While individual training remains essential, organizational leverage through modeling of norms and setting of expectations at the unit level may be particularly effective in hierarchical climates. Specifically, high power distance in Chinese hospitals may amplify the influence of leadership on infection prevention climate, while collectivism could strengthen peer-driven norms. These cultural dynamics might limit the generalizability of sequential mediation effects to low-power-distance or individualistic contexts. Future research should explicitly compare these mechanisms across cultural contexts to refine cross-national intervention strategies.

## 5 Study limitations

This study has several limitations. First, the cross-sectional design limits causal inference and precludes firm conclusions about temporal ordering. To address this, the hypothesized directions were grounded in established theory and examined using validated analytical procedures. In addition, we carefully avoided causal terminology throughout the manuscript to ensure consistency with the study design. Second, the use of self-reported data raises the risk of social desirability and common method bias. To minimize these risks, we employed validated scales, assured respondent anonymity, and included reverse-coded items, although such bias cannot be entirely ruled out. Third, important contextual variables such as workload, staffing ratios, and leadership styles were not included. These factors may influence both safety climate and compliance behaviors, and their omission reduces the comprehensiveness of the model. Future studies should incorporate these variables to provide a more holistic understanding. Finally, longitudinal or cluster-randomized designs are needed to test the hypothesized pathways, while mixed-methods approaches (e.g., interviews or ethnographic observations) could further illuminate the social and cultural dynamics underlying infection control practices. Developing validated unit-level diagnostic tools for infection prevention climate would also enable real-time monitoring and targeted interventions.

## 6 Conclusion

In conclusion, our study demonstrates that unit-level infection prevention climate is significantly associated with nurses' compliance with standard precautions, both directly and via attitudinal mediation. Infection prevention climate is a distinct and actionable target, complementary to individual-level interventions, and especially relevant in culturally hierarchical healthcare settings. Integrated strategies that combine system-level climate improvements with interventions enhancing personal conviction are likely to yield the most sustainable compliance gains. These findings provide guidance for hospital administrators and policymakers aiming to optimize infection prevention efforts and improve patient safety outcomes.

## Data Availability

The original contributions presented in the study are included in the article/Supplementary material, further inquiries can be directed to the corresponding author.
