# Supplementary material for: A serial mediation model of patient safety climate on nurses' compliance with standard precautions: the roles of infection prevention climate and attitude
Source: Front Public Health. 2025 Sep 18;13:1673026. doi: 10.3389/fpubh.2025.1673026 (PMC12489939; doi:10.3389/fpubh.2025.1673026)
Supplement: Supplementary file 1 [file Table_1.pdf]

Supplementary Table S1 Model Comparison Between Final and Constrained Models

Model	χ^2 (df)	CFI	TLI	RMSEA	SRMR	AIC	BIC	R^2(IPC)	R^2(COMP)
Excluding PSC	58.7(10)	0.892	0.875	0.071	0.051	1389.7	1421.5	0.218	0.412
Final model	32.1(10)	0.941	0.927	0.052	0.038	1256.3	1302.1	0.437	0.573

Model fit thresholds: CFI/TLI>0.90, RMSEA<0.08, SRMR<0.05; Key differences: ΔAIC=+133.4, ΔR^2(IPC)=-0.219; All χ^2 tests significant at $p<0.001$
